# Switching to a Healthy Diet Prevents the Detrimental Effects of Western Diet in a Colitis-Associated Colorectal Cancer Model

**DOI:** 10.3390/nu12010045

**Published:** 2019-12-23

**Authors:** Charlotte Gröschel, Maximilian Prinz-Wohlgenannt, Ildiko Mesteri, Sobha Karuthedom George, Lena Trawnicek, Denise Heiden, Abhishek Aggarwal, Samawansha Tennakoon, Maximilian Baumgartner, Christoph Gasche, Michaela Lang, Rodrig Marculescu, Teresa Manhardt, Martin Schepelmann, Enikö Kallay

**Affiliations:** 1 Institute of Pathophysiology and Allergy Research, Center for Pathophysiology, Infectiology and Immunology, Medical University of Vienna, 1090 Vienna, Austria; groeschel.charlotte@gmail.com (C.G.); prinz-wohlgenannt@gmx.at (M.P.-W.); sobha.karuthedom@gmail.com (S.K.G.); lena.trawnicek@inode.at (L.T.); denise.heiden@meduniwien.ac.at (D.H.); 1988.abhishek@gmail.com (A.A.); samawansha_10@yahoo.com (S.T.); teresa.manhardt@meduniwien.ac.at (T.M.); martin.schepelmann@meduniwien.ac.at (M.S.); 2 Institute of Pathology Überlingen, 88662 Überlingen, Germany; ildiko.mesteri@hotmail.com; 3 Division of Gastroenterology and Hepatology, Department of Internal Medicine 3, Medical University of Vienna, 1090 Vienna, Austria; maximilian.baumgartner@meduniwien.ac.at (M.B.); christoph.gasche@meduniwien.ac.at (C.G.); lang@microbial-ecology.net (M.L.); 4 Department of Laboratory Medicine, Medical University of Vienna, 1090 Vienna, Austria; rodrig.marculescu@meduniwien.ac.at

**Keywords:** inflammatory bowel disease, colitis-associated cancer, microbiome, western diet, vitamin D, non-alcoholic fatty liver disease, CYP24A1, Wnt pathway, aberrant crypt foci, mucosal regeneration

## Abstract

Inflammatory bowel disease increases the odds of developing colitis-associated cancer. We hypothesized that Western-style diet (WD) aggravates azoxymethane (AOM)/dextran sulfate sodium salt (DSS)-induced colitis-associated tumorigenesis and that switching to the standard AIN93G diet will ameliorate disease symptoms even after cancer initiation. Female BALB/c mice received either WD (WD group) or standard AIN93G diet (AIN group) for the whole experimental period. After five weeks, the mice received 12.5 mg/kg AOM intraperitoneally, followed by three DSS cycles. In one group of mice, the WD was switched to AIN93G the day before starting the first DSS cycle (WD/AIN group). Feeding the WD during the whole experimental period aggravated colitis symptoms, shortened the colon (*p* < 0.05), changed microbiota composition and increased tumor promotion. On molecular level, the WD reduced proliferation (*p* < 0.05) and increased expression of the vitamin D catabolizing enzyme *Cyp24a1* (*p* < 0.001). The switch to the AIN93G diet ameliorated this effect, reflected by longer colons, fewer (*p* < 0.05) and smaller (*p* < 0.01) aberrant colonic crypt foci, comparable with the AIN group. Our results show that switching to a healthy diet, even after cancer initiation is able to revert the deleterious effect of the WD and could be an effective preventive strategy to reduce colitis symptoms and prevent tumorigenesis.

## 1. Introduction

Incidence of inflammatory bowel diseases (IBD) is increasing continuously. Standard therapies for ulcerative colitis and Crohn´s disease have limited efficacy [1]. The increasing incidence of IBD in industrialized regions of the world and the new onset in countries that are adopting a Western lifestyle suggest that a change in nutritional habits to a typical Western diet contribute to IBD [2,3]. IBD patients are at increased risk of developing inflammation-associated colorectal cancer (CAC) [4]. A recent meta-analysis reported that tumorigenesis in IBD patients occurs rather in the proximal colon and is associated with a worse prognosis compared with sporadic colorectal cancer (CRC) [5]. There is some evidence that proximal colon tumors develop more often in women [6].

Epidemiological data suggest a positive correlation between high fat diets and increased incidence of IBD and CRC [7,8]. Animal-derived fat is a risk factor for colon cancer, whereas adequate amounts of n-3-polyunsaturated fatty acids, which are found in soybean oil [9], decrease the risk to develop CRC [10] and ameliorate inflammation [11,12]. The total amount of dietary fat and the source and composition of the lipids influence tumorigenesis and inflammatory processes [13]. Saturated fatty acids have pro-inflammatory properties [14]. Butter fat promotes intestinal inflammation via increase of intestinal Tumor Necrosis Factor-α production in mice [15]. The release of inflammatory cytokines and the induction of oxidative stress from innate and adaptive immune cells activate β-catenin and nuclear factor kappa-light-chain-enhancer of activated B cells (NF-κB), two transcription factors that regulate intestinal epithelial wound healing but also lead to malignant transformation [16,17,18].

Calcium and vitamin D insufficiency are common in industrialized countries and might be implicated in the etiology of numerous chronic diseases [19]. Feeding mice a diet high in fat but low in calcium, vitamin D, folate, and fiber, activated transcription of signaling molecules and target genes of the Wnt/β-catenin pathway and caused colonic tumor formation after two years. Supplementation with 7 mg/g calcium and 2300 IU vitamin D prevented tumor formation and activation of the Wnt pathway [20,21]. Feeding a methyl-donor-deficient diet to rats increased the inflammatory response to dextran sulfate sodium salt (DSS) treatment [22]. On the molecular level, Ca^2+^ and vitamin D target several hallmarks of cancer [23,24,25]. Our group has shown that Ca^2+^ exerts anti-proliferative properties through the calcium-sensing receptor (CaSR) [26]. In vitro and in vivo studies indicate that both calcium and vitamin D are crucial in the maintenance of intestinal barrier function and that they have anti-inflammatory properties [27,28,29]. Ca^2+^ and vitamin D act synergistically leading to differentiation and inhibition of proliferation by modulating the Wnt/β-catenin pathway [30,31]. Although there is evidence from in vitro and animal studies that calcium and vitamin D could counteract the pathogenesis of IBD through several mechanisms, there is still no clear dietary advice for IBD patients due to lack of interventional studies proving efficacy of food components for disease prevention and therapy [32]. 

Chemical induction of inflammation-induced tumorigenesis by azoxymethane (AOM) and DSS is a model which resembles human CAC in terms of histopathological and inflammatory features [33]. DSS-induced tissue damage preferentially affects the proliferative crypt compartment, the source of epithelial regeneration [34]. 

We investigated whether the detrimental effect of a so-called “Western-style diet” (WD), high in animal fat but deficient in calcium, vitamin D, methyl donors, and fiber, on colitis-associated tumor formation can be prevented by switching the WD to the normal “healthy” AIN93G diet, containing soybean oil and adequate levels of micronutrients and fiber. 

## 2. Materials and Methods 

### 2.1. Animals, Diets, and the AOM/DSS Tumorigenesis Model

Four-week old female BALB/c mice (Charles River, Germany) were housed at the animal facility of the Institute of Pathophysiology and Allergy Research of the Medical University of Vienna in a controlled environment with 12 hours light–dark cycle. Guidelines of the European Union Regulations on Care and Use of Laboratory Animals were followed when maintaining the living conditions and performing the experiments. The study was approved by the Ethics Committee of the Medical University of Vienna as well as the Austrian Federal Ministry for Science, Education, and Research (No: BMWF-66.009/0069-WF/V/3b/2015). Upon delivery, the mice were randomly divided into three groups and were acclimatized for 14 days on a regular chow, before they received either the AIN93G diet or the WD (ssniff EF R/M acc. TD88137 mod.) modified for fat, calcium, vitamin D, methyl donors, and fiber, similar to the New Western Diet from Newmark et al. [20] (Table S1). 

**Figure 1:**
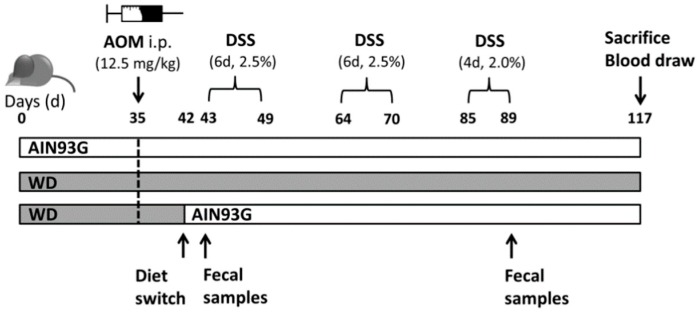
Graphical representation of the treatment protocol. Three groups of female BALB/c mice received either the AIN93G diet or the Western-style diet (WD) for 35 days, before they received azoxymethane (AOM) intraperitoneal (i.p.) one day before the start of the first cycle of dextran sulfate sodium salt (DSS), in one group, the WD was switched to the AIN93G diet. The mice were sacrificed 28 days after the third cycle of DSS.

One group received exclusively the AIN93G diet (AIN group, eight animals) while another received only the WD (WD group, nine animals) throughout the experimental period. The third group was maintained on the WD for 42 days before the diet was switched to the AIN93G diet (WD/AIN group, eight animals) one day before the start of the first DSS cycle (MP Biomedicals, Solon, OH, USA). After 35 days feeding either WD or AIN93G diet, the mice received 12.5 mg/kg AOM (Sigma Aldrich, St. Louis, MO, USA) via intraperitoneal (i.p.) injection to induce tumorigenesis. The mice were treated with three cycles of DSS (first and second cycle DSS: Six days, 2.5% per cycle, third cycle: Four days, 2% DSS), added to autoclaved tap water to induce inflammation for tumor promotion. The mice were euthanized 28 days after the end of the third DSS cycle, on day 117 (Figure 1). Blood was collected via heart puncture. Kidney, liver, spleen, and colon were removed. The colon was rinsed in ice–cold phosphate-buffered saline (PBS) and 0.5 cm from each end was cut and snap frozen in liquid nitrogen. The remaining colon was rolled into a Swiss roll [35], fixed in 4% formaldehyde-PBS, and subsequently paraffin-embedded. Livers and kidneys were cut into two halves for snap freezing and paraffin-embedding as described above.

### 2.2. Analysis of the microbiota

Feces were collected on the first day of the first and on the last day of the third DSS cycle. Samples were snap frozen in liquid nitrogen and stored at −20 °C. DNA was isolated using the QIAamp Fast DNA stool Mini kit (Qiagen) according to the manufacturer’s instructions, with bead beating using Lysing Matrix E tubes (MP Biomedicals) before extraction. 16S rRNA gene amplicon sequencing and library preparation was performed using a standard Illumina protocol [36]. Reads were processed using the software packages DADA2 [37] and SINA [38]. For the analysis of sample similarity modified Rhea scripts were used [39]. Generalized UniFrac distances were visualized using multi-dimensional scaling [40]. We assessed cluster significance using permutational multivariate analysis of variance. Testing for significant differences in diversity and bacterial abundances was performed using Kruskal-Wallis Rank Sum Test with Benjamin-Hochberg method for correction for multiple comparisons. We used the Mann-Whitney U test to compare phylogenetic distances.

### 2.3. Tissue Samples, RNA Isolation, Reverse Transcription and Quantitative RT-PCR 

Snap frozen tissue was homogenized with the Precellys 24-Dual Homogenizer (Precellys, France) in TRIzol reagent (Life technologies, USA) and RNA was isolated according to the manufacturer´s instructions. RNA integrity was verified on agarose gels stained with Gel Green (Peqlab, Austria). Reverse transcription and qRT-PCR was performed as described before [41]. For calculation according to the ∆∆C_t_ method, the target gene expression was normalized to two reference genes, mouse beta-actin (*ActB*) and mouse eukaryotic translation elongation factor 1 beta 2 (*Eef1β2*), and was set relative to a total RNA calibrator (Clontech, Mountain View, CA, USA). Primer sequences of the reference genes [42] and of *Occludin* and *Tlr4* [27] have been described previously. Primer sequences of the other genes of interest are shown in Table S2**.**

### 2.4. Histological Examination of Colon Sections 

Four-micron tissue sections were cut from paraffin-embedded colonic Swiss rolls and were stained with Mayer´s Hematoxylin Solution and Eosin (Sigma Aldrich). A pathologist evaluated the chronic inflammation and tumorigenesis blinded for the treatments and evaluated the degree of colitis based on the number of lesions as well as their severity, assigning a histopathological score (0–4), 0 = no colitis, 4 = severe colitis [43]. Aberrant crypt foci (ACFs), dysplasia, and carcinoma in situ were diagnosed and the size of the lesions was quantified by counting the affected crypts. The dysplasia score was determined according to the method by Riddell et al. [44].

### 2.5. Immunohistochemistry and Immunofluorescence

The Swiss rolls were deparaffinized and rehydrated. For antigen retrieval, slides were incubated in 95 °C citrate buffer (pH = 6) for 20 min. The slides were incubated with the first antibody overnight (mouse monoclonal anti-Ki-67, Origene, MKI67, clone UMAB107) 1:750 in 0.1% goat serum in PBS-T. The HRP/antiMOUSE DAKO Envision System Kit (Dako, K4000) was used to visualize the antigen. three regions of two to five intact untransformed healthy colonic crypts per mouse intestine were chosen, the number of Ki-67-positive and Ki-67-negative cells were counted in a blinded fashion and the percentage of Ki-67-positive cells were calculated. The area of lymph follicles was measured by HistoQuest Software (TissueGnostics, Vienna, Austria). Immunofluorescence staining was performed as described previously [41]. For vitamin D receptor (VDR) immunofluorescence staining of the colon, the tissue slides were incubated with the primary antibody (VDR, SAB4503071, Sigma Aldrich, Austria) 1:100 in PBS-T for one hour at RT. The secondary antibody (Dylight 549 anti-IgG antibody, Vector Laboratories, Peterborough, UK) was incubated 1:1000 for one hour in 0.05% TBS-T at RT. Nuclei were stained with DAPI (Roche, Vienna, Austria) for 10 min and slides were mounted with Fluoromount-G (Southern Biotech, Birmingham, AL, USA). Whole slide images of Swiss rolls were acquired using TissueFAXS hard- and software (TissueGnostics). 

### 2.6. Statistical Analysis

All statistical analyses were performed with SPSS version 22 (IBM, USA) and graphs were drawn by GraphPad Prism version 7 (GraphPad Software Inc., San Diego, USA). Non-normally distributed data were log transformed to achieve normal distribution and analyzed by one-way ANOVA with Tukey post-hoc test, where appropriate. Significant outliers were detected by Grubbs‘ outlier test and were excluded from analysis. Non-normally distributed data were analyzed by Kruskal Wallis with Dunn´s post-hoc test. 

## 3. Results

### 3.1. The WD Negatively Affects Body Weight, Colon Length, and Liver and Spleen Weight

**Figure 2:**
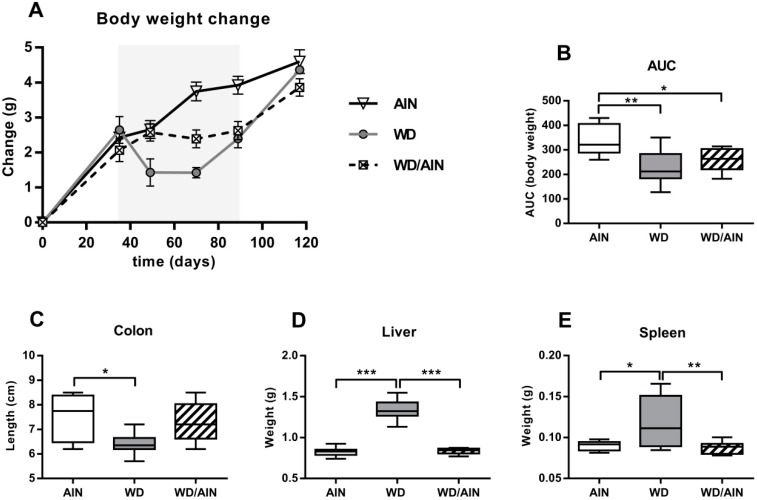
Effect of the WD on macroscopic changes (**A**) Body weight loss during the period of AOM/DSS administration (grey-shaded area). Day 0: Start of AIN93G or WD feeding, day 35: AOM i.p., day 49: End of first DSS cycle, day 70: End of second DSS cycle, day 89: End of third DSS cycle, and day 117: Day of sacrifice. Body weight of each mouse was set relative to day 0. Mean ± SEM, (**B**) Body weight changes based on the analysis of the area under the curve (AUC) during the whole experimental period of 117 days. (**C**) Effect of the diet on colon length, (**D**) liver, and (**E**) spleen weight measured on the day of sacrifice. *n* = 8, median, interquartile range, and whiskers (min to max); ANOVA with Tukey post-hoc test; * *p* < 0.05, ** *p* < 0.01, and *** *p* < 0.001.

After the first five weeks of feeding the test diets, i.e., at the time of AOM administration the body weight gain was similar in all three diet groups (Figure 2A), although the energy content of the WD was roughly 15% higher. After AOM administration, the mice fed the AIN93G diet gained weight continuously throughout the whole experiment, despite the AOM/DSS treatment. AOM/DSS administration led to a significant initial body weight loss in the WD group compared with the AIN group, which persisted during the experimental period from the day of AOM injection until the end of the third cycle of DSS (Figure 2A, 33%, *p* < 0.01). After the end of the second DSS cycle, the WD group started to gain weight despite a third exposure to 2% DSS for four days. In the WD/AIN group, the weight of the animals stagnated during the AOM/DSS administration, with an area under the curve (AUC) significantly different from the AIN group (Figure 2B, 23%, *p* < 0.05), starting to grow only after the last DSS cycle. 

At the end of the experiment, all animals, irrespective of the diet received had a similar gain in weight compared with the weight on day 0 (Figure 2A). From the day of the AOM injection till the end of the third DSS cycle most of the mice receiving the WD either lost weight or the weight remained constant (Figure S1). 

Feeding the WD led to a significant shortening of the colon, a marker for the severity of inflammation, compared with the colons of mice receiving the AIN93G diet (Figure 2C, 15%, *p* < 0.05), while the diet switch prevented this shortening (Figure 2C).

Inflammation often leads to increase in liver and spleen weight. Indeed, the liver of the mice fed the WD were significantly heavier compared with the liver of the animals in the two other diet groups (Figure 2D, 1.6-fold, *p* < 0.001 versus AIN group; 1.6-fold, *p* < 0.001 versus WD/AIN group). The spleen weight in the WD group was 1.3-fold higher compared with the AIN group (Figure 2E, *p* < 0.05) and 1.4-fold higher than in the WD/AIN group (Figure 2E, *p* < 0.01). In the WD group, one mouse had to be euthanized before the end of the experimental period and was not included in the analysis.

Interestingly, in mice under the same dietary regime for 117 days but not exposed to AOM/DSS, WD has not caused any changes in body weight (Figure S2A), or colon length (Figure S2B). The number of colonic lymph follicles (Figure S2C) and the spleen weight (Figure S2D) remained also unaffected. However, feeding exclusively the WD significantly increased liver weight (Figure S2E).

### 3.2. The WD Increases the Number and Size of Colonic Lymph Follicles and Premalignant Lesions 

**Figure 3:**
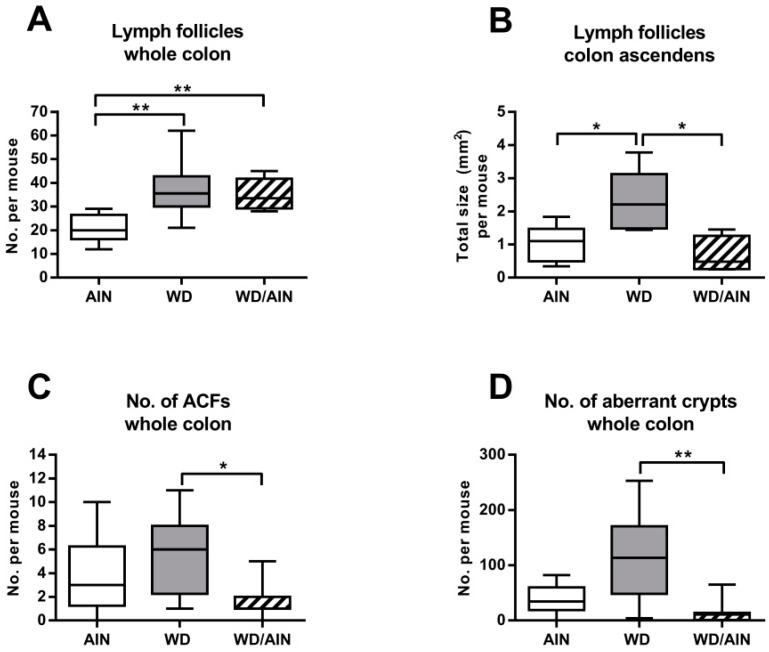
Effect of the diet on the (**A**) number (*n* = 7–8), and (**B**) size of colonic lymph follicles (*n* = 4–5), ANOVA with Tukey post-hoc test. Effect of the diet on (**C**) number of aberrant crypt foci (ACFs), ANOVA with Tukey post-hoc test on log transformed data (*n* = 7–8). Effect of the diet on the (**D**) number of aberrant crypts (*n* = 7–8), Kruskal Wallis with Dunn post-hoc test; median, interquartile range, and whiskers (min to max); * *p* < 0.05, ** *p* < 0.01.

In our chemically induced colon cancer model, feeding the WD significantly increased the number of colonic lymph follicles (Figure 3A, 1.8-fold versus AIN, *p* < 0.01) and switching to the AIN93G diet could not reverse this effect (Figure 3A, 1.7-fold versus AIN, *p* < 0.01). The total size of lymph follicles per colon was similar among the groups (data not shown). However, in the ascending colon, the lymph follicles were significantly larger in the WD group, when compared both with the AIN group (Figure 3B, 2.3-fold, *p* > 0.05) and the WD/AIN group (Figure 3B, 3.4-fold, *p* < 0.05). In the distal colon, the size of lymph follicles did not change (data not shown). The histopathological score of chronic inflammation was not different among the groups (Figure S3A).

#### WD promotes ACF formation.

We evaluated the number and size of mucosal regions forming ACF, the earliest premalignant lesions that precede dysplasia in sporadic CRC as well as in CAC. ACF can be used as a surrogate marker to verify the effect of diet on colonic tumorigenesis [45,46,47]. The WD led to a 3.2-fold increase in the number of ACF (Figure 3C, *p* < 0.05) and to an increase in the size of all ACF, which was reflected by a 10.3-fold higher number of aberrant crypts forming the ACF per mouse (Figure 3D, *p* < 0.01) compared with animals which were switched to the AIN93G diet. There was no significant difference between the AIN and WD/AIN groups. We found five times more aberrant crypts in the ascending colon than in the descending colon (data not shown).

Feeding exclusively the “healthy” AIN93G diet prevented the development of high-grade dysplasia and of carcinoma in situ (Table 1). 

The area of mucosa transformed to high-grade dysplasia and to carcinoma in situ (described as total number of aberrant colonic crypts) was highest in the WD group, and the diet switch reduced their size (Table 2). Moreover, the loss of normal crypt architecture was most severe in the WD group, while the AIN group had the least disturbed crypt architecture, albeit these differences were not significant (Figure S3B).

### 3.3. Effect of the WD on Gene Expression in the Liver 

**Figure 4:**
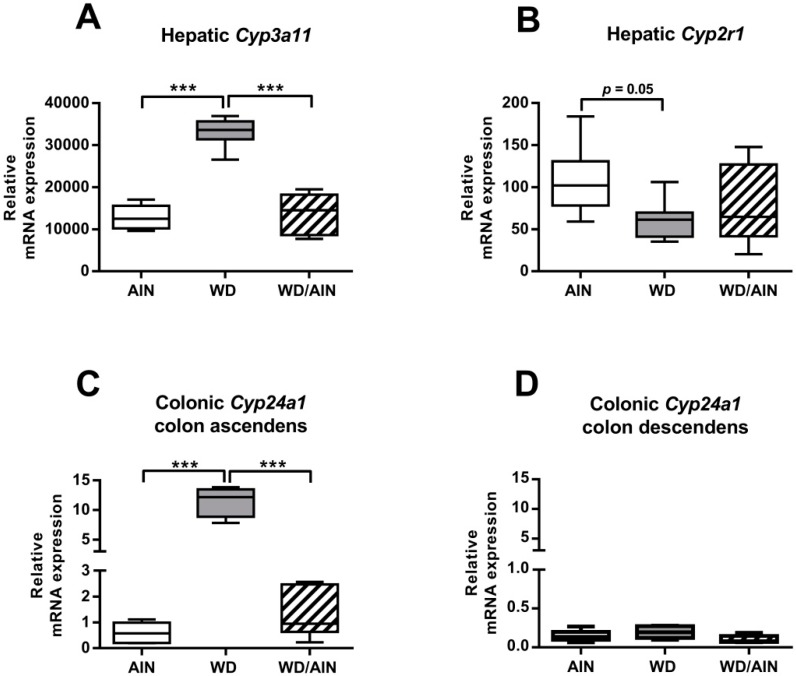
Effect of the diet on mRNA expression of the hepatic (**A**) *Cyp3a11*, and (**B**) *Cyp2r1* and on the colonic expression of *Cyp24a1* in (**C**) the ascending and (**D**) descending colon (*n* = 4–7). Median, interquartile range, and whiskers (minimum to max). In the WD group of (**A**,**C**) one outlier, as determined by Grubbs’ test, was excluded from the analysis. ANOVA with Tukey post-hoc test; *** *p* < 0.001.

As AOM is metabolized in the liver, it also could affect liver function [48], therefore we tested the effect of the diet on the functionality of the liver in general by measuring the mRNA expression of the bile acid detoxifying enzyme *Cyp3a11* and its nuclear receptor the pregnane X receptor (*Pxr*). Feeding exclusively the WD led to a 2.6-fold increase of *Cyp3a11* when compared with the animals fed with the AIN93G diet, and 2.4-fold higher (Figure 4A, *p* < 0.001) when compared with the WD/AIN group, without affecting *Pxr* expression (data not shown).

Then, we analyzed the mRNA expression level of the vitamin D system. This is composed of CYP2R1 and CYP27A1, responsible for the hydroxylation of vitamin D on position 25 into the precursor 25-D_3_, CYP27B1, the enzyme responsible for transforming 25-D_3_ into the active hormone 1,25-D_3_ and the vitamin D catabolizing enzyme CYP24A1, and the vitamin D receptor (*Vdr*), the transcription factor needed by 1,25-D_3_ to exert its genomic effects [49]. The mRNA levels of *Cyp2r1* appeared to be decreased in the WD group when compared with the AIN group (Figure 4B, 43.62%, *p* = 0.05). The mRNA levels of the hepatic *Vdr* and *Cyp24a1* were below the detection limit. The WD had no significant effect on the hepatic mRNA expression of *Cyp27a1* and *Cyp27b1* (data not shown).

### 3.4. The WD Changed the Composition of the Microbiota

Diet and liver function have a strong impact on the microbiota. Stool samples were collected at day 43 (start of the first DSS cycle and first day of diet switch in the WD/AIN group) and day 89 (end of the third DSS cycle and 47 days after switching the diets in the WD/AIN group, see Figure 1). 

**Figure 5:**
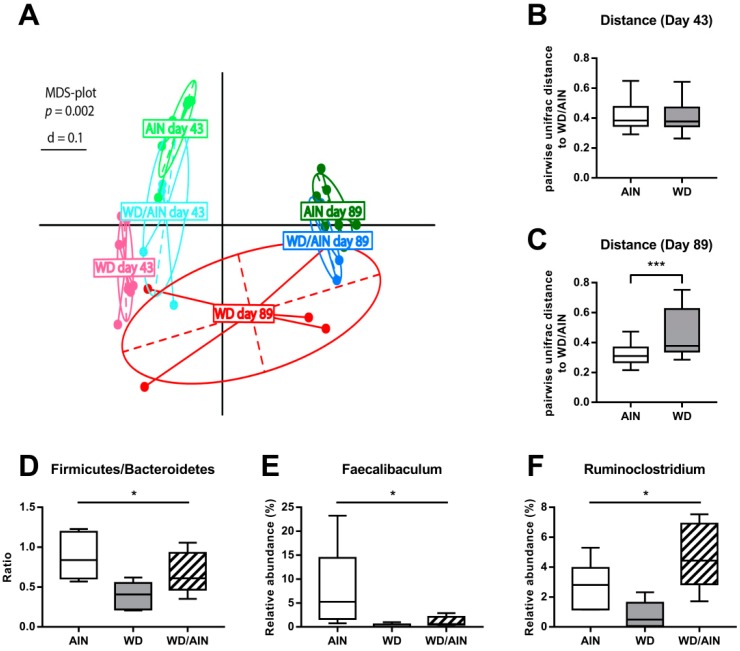
Effect of the diets on the microbiome. (**A**) Multi-dimensional scaling plot of fecal samples from day 43 (first day after diet switch) and from day 89 (47th day after diet switch), (**B**,**C**) pairwise UniFrac distances between the WD/AIN group and the AIN and WD groups, respectively. Effect of the diets on day 89 on the: (**D**) Firmicutes/Bacteroidetes ratio, (**E**) relative abundance of Faecalibaculum, and (**F**) relative abundance of Ruminoclostridium. * *p* < 0.05, *** *p* < 0.001.

At both time points the microbiota composition was different between the AIN, WD, and WD/AIN groups as indicated by significant clustering in the MDS plot (Figure 5A, *p* = 0.002). Pairwise UniFrac distances between different groups allowed us to quantify the differences in microbiota composition [40]. On day 43, before induction of inflammation, on the first day of diet switch, all three diet groups clustered close to each other (Figure 5B). The chronic inflammation due to the DSS cycles affected profoundly the microbiota composition. On day 89, after 47 days of chronic inflammation, the microbiota composition in the AIN and WD/AIN group, was still similar (Figure 5C, *p* < 0.001) and significantly different from the composition of the WV group. The WD decreased Firmicutes/Bacteroidetes ratio (Figure 5D, *p* = 0.05) and the relative abundance of the bacterial genera *Faecalibaculum,* which was only detectable in three out of five samples of the WD group while present in all samples from the AIN and WD/AIN group (Figure 5E, *p* = 0.05). Feeding the WD decreased also the relative abundance of the bacterial genera *Ruminoclostridium* (Figure 5F, *p* = 0.05).

### 3.5. Effect of the WD on the Vitamin D System in the Colon and on Major Pathways Affecting the Colonic Crypt Architecture

We assessed the effect of the diet separately in the ascending and descending colon. In ascending colons of the WD group, the expression of *Cyp24a1* was 18.7-fold (Figure 4C, *p* < 0.001) and 8.8-fold (Figure 4C, *p* < 0.001) higher when compared with the AIN group and the WD/AIN group, respectively, although the *Vdr* mRNA level did not change significantly (Table S3), similar to *Cyp27b1* or *Casr* expression (Table S3). In colon descendens, the diet had no significant effect on the mRNA expression of the investigated genes (Figure 4D, Table S3). The VDR protein was highly expressed in all samples and the number of VDR-positive crypt cells was similar in all diet groups, suggesting that the process of inflammation does not affect VDR expression (Figure S4A and S4B).

**Figure 6:**
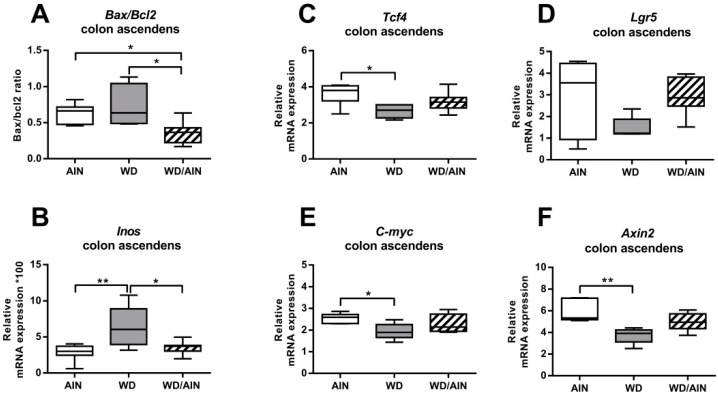
Effect of the diet on the mRNA expression of genes involved in apoptosis, survival, inflammation (**A**,**B**) and of genes of the Wnt pathway (**C**–**F**) in colon ascendens. *n* = 4–8 (**A**–**E**), *n* = 5–8 (**F**) median, interquartile range, and whiskers (min to max); ANOVA with Tukey post-hoc test (**A**–**E**) or Kruskal Wallis with Dunn post-hoc test (F), * *p* < 0.05, ** *p* < 0.01.

**Figure 7:**
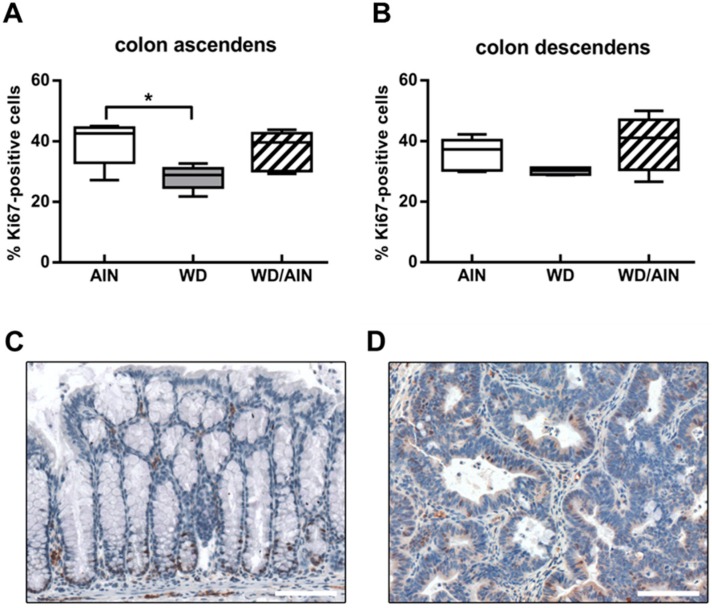
Effect of the diet on proliferation of colonic crypts. Ki67-positive cells in untransformed crypts of (**A**) colon ascendens and (**B**) descendens. For each animal, three regions consisting of two to five crypts were chosen and the total number of Ki67-positive and of Ki67-negative cells were determined. *n* = 4–6; median, interquartile range, and whiskers (min to max); in the WD group of (**A**) one outlier, as determined by Grubbs’ test, was excluded from analysis; and ANOVA with Tukey post-hoc test, * *p* < 0.05. Representative pictures of Ki67 (brown) staining (**C**) in the untransformed mucosa of the WD/AIN group and (**D**) in a tumor of the WD group. Color and contrast of the images (**C**,**D**) were non-linearly enhanced for purpose of presentation only, scale bars = 100 µM.

Crypt architecture is controlled by the balance between apoptosis and proliferation. The canonical TCF4/β-catenin-dependent Wnt pathway plays a central role in regulating colonic crypt architecture [50]. We compared the effect of the diet on the mRNA expression of relevant genes involved in apoptosis, the Wnt pathway, and numerous inflammatory markers in the ascending and descending colon of the mice (Figure 6, Table S3). We assessed the number of Ki67-positive colonic crypt cells as a marker of proliferation (Figure 7). We observed most of the changes in colon ascendens, while the effect in colon descendens was much less evident (Table S3).

We investigated the effect of the WD on the balance between expression levels of anti- and pro-apoptotic proteins. In all groups, the *Bax/Bcl2* ratio was below one, indicative of higher survival capacity. In colon ascendens, the diet switch decreased the *Bax/Bcl2* ratio, when compared with the AIN group (43.32%, *p* < 0.05), or the WD group (49.90%, *p* < 0.05) (Figure 6A), suggesting less apoptosis and higher epithelial recovery. The lowest expression of the anti-apoptotic gene *Bcl-2* was in the WD group (Table S3, *p* < 0.05). 

Sucrase isomaltase, a marker of differentiation, slightly decreased in colon ascendens of the WD group (36.68%, *p* = 0.05), and remained low in the group switched to the AIN93G diet (39.43%, *p* < 0.05) (Table S3).

In order to assess the effect of the WD on mucosal regeneration, we analyzed the extent of the proliferative zone in intact untransformed colonic crypts by counting the number of Ki-67-positive and Ki-67-negative cells (Figure 7). 

Unexpectedly, the WD decreased significantly the proliferative zone of the colonic crypts in the colon ascendens, reducing the number of Ki-67-positive cells by 28.8% (*p* < 0.05) compared with the AIN group. Even the tumor region of the group receiving exclusively the WD had lower levels of Ki-67 compared with the WD/AIN group (Figure 7).

The inducible nitric oxide synthase (iNOS), a target of NF-κB [51,52] is highly expressed in colon mucosa of IBD patients [53]. Feeding exclusively the WD increased *iNOS* 2.2-fold (*p* < 0.01) compared with the AIN group and 1.8-fold compared with the WD/AIN group (*p* < 0.05) (Figure 6B). 

The Wnt pathway is a master regulator of the colonic architecture [54]. In colon ascendens, feeding the WD diet throughout the experimental period led to a significant reduction in mRNA levels of many molecules involved in the canonical pathway, indicating a generally reduced activation state of the Wnt pathway. The expression level of the Wnt transcription factor *Tcf4* was lowest in the WD group (Figure 6C, 26.7%, *p* < 0.05) similarly with the Wnt activating co-receptor and Wnt target gene *Lgr5*, although this did not reach statistical significance (Figure 6D). WD decreased the expression of *C-myc* (Figure 6E, 23.9%, *p* < 0.05) and of *Axin2* (Figure 6F, 26.8%, *p* < 0.01), targets of the Tcf4/β-catenin transcription factor complex. Other genes known to be activated by Tcf4/β-catenin were also decreased by the WD, such as *CD44* and *Cyclin D1* (Table S3), but without reaching statistical significance. In the WD group, we observed a slight increase of mRNA levels of the Wnt-inhibitor *Dkk1*, although this did not reach significance either (Table S3). The diet switch decreased mRNA levels of *Gsk-3β*, which phosphorylates β-catenin for consecutive degradation [55], by 29.1% (Table S3, *p* < 0.001). β-catenin was localized mainly in the membrane and we have not observed nuclear localization in any of the dietary groups using immunofluorescence staining (data not shown).

In the descending colon, most of the analyzed genes were not affected by the diet (Table S3). Among the analyzed genes of the Wnt pathway, only *Lgr5*, the intestinal stem cell marker which is expressed at the base of the crypts [56], was significantly higher expressed when compared with the WD/AIN group (Table S3, *p* < 0.05). Occludin, a transmembrane tight junction protein, which regulates the permeability of macromolecules, is downregulated in the colons of IBD patients [57,58]. *Occludin* expression was highest in mice fed exclusively the AIN93G diet: 1.8 times higher than in the WD/AIN group (*p* < 0.01) and 1.4 times higher than in the WD group (Table S3). 

## 4. Discussion

We found that a diet high in saturated fat derived from butter, but low in calcium, vitamin D, methyl donors, and fiber worsened chemically-induced colitis and tumor formation in mice and changed the composition of the microbiota. We are the first to show that a switch to the normal “healthy” rodent diet before DSS administration, and thus induction of colonic inflammation, ameliorated the symptoms of chronic inflammation and tumor promotion. The mice fed the WD developed not only low-grade dysplasias but also high-grade dysplasias and carcinomas, however switching the diet from WD to the normal AIN93G chow prevented the growth of these lesions or promoted the repair/healing processes. Feeding exclusively the healthy diet prevented the advance of the low-grade dysplasias into more aggressive lesions. The length of the colons, the weight of the livers and spleens, and the size of colonic lymph follicles in the ascending colons of the animals that underwent the diet switch were all comparable with the values measured in the mice receiving exclusively the healthy AIN93G diet, indicating the effectiveness of the diet change. Interestingly, although the histopathological inflammation score and the extent of destruction of mucosal crypt architecture were similar among the groups, the mice in the AIN and in the WD/AIN groups had less severe diarrhea and clinical symptoms, lost less weight and recovered faster after the DSS administration. 

In our CAC model, the diet affected mainly the mucosa of the proximal colon, which was also the major site of tumorigenesis, similar to previous studies [59]. There is evidence that diet affects tumorigenesis in the different colonic segments differently [60] and tumors of the ascending colon are clinically, pathologically, and prognostically different from the tumors of the descending colon [6]. 

The AOM/DSS model is widely used for studying colitis-associated cancer. AOM causes the genetic injury and the constant regeneration needed after the DSS-induced tissue injury is the promoting impulse for tumorigenesis [59]. Feeding the mice the WD perturbed the regeneration process, mainly in the ascending colon by reducing proliferation and expression of Wnt target genes. These findings were unexpected, as several studies reported the opposite [61,62]. Nevertheless, in a recent study in DSS-treated mice, reduced mucosal proliferation promoted the development of colonic dysplasia [63]. Our group has also shown that feeding a low vitamin D diet increased tumorigenesis in an AOM/DSS mouse model but simultaneously decreased expression of Ki67 in untransformed colonic mucosa [64]. The severity of IBD seems to correlate with vitamin D deficiency [65] and low levels of vitamin D are a feature of Western diet [66]. However, it is probably never a single nutrient of importance, but the interaction among several nutrients is more effective in counteracting IBD and tumorigenesis.

A further effect of the WD was the significant increase in the expression of *Cyp24a1*, the vitamin D degrading enzyme, in the ascending colon. Higher CYP24A1 levels reduce the local availability of 1,25-D_3_ and thus impair its anti-inflammatory action. This might be a further explanation for how a WD promotes colorectal tumorigenesis, as CYP24A1 expression is known to be increased in colorectal tumors [24,67], and we have shown previously that overexpression of CYP24A1 increased aggressiveness of human colorectal cancer cells in a xenograft model [68]. 

The loss of body weight, a marked shortening of the colon, higher spleen weight [69], and the increase in lymph follicles, upon feeding the WD are indicative of severe colonic inflammation [70] and unresolved chronification of the inflammatory process [71]. It has been shown previously that methyl donor-deficiency promotes fat liver development [72] and indeed the WD led to heavier livers. There is evidence that non-alcoholic fatty liver disease (NAFLD) and severity of IBD are interconnected [73]. Notably, NAFLD patients develop rather proximal colorectal tumors [74]. 

The WD increased significantly the expression of the hepatic *Cyp3a11*, the murine homolog of human CYP3A4, one of the key enzymes involved in drug metabolism [75] and in bile acid detoxification [76,77]. A recent study also showed that diets rich in saturated fat increase murine *Cyp3a11* levels [78,79]. The diet switch was able to prevent the increase of *Cyp3a11* an important observation for the treatment of IBD, because many drugs applied in IBD are metabolized by CYP3A4 [80]. 

The pro-inflammatory and pro-tumorigenic effect of the WD might be due in part to the low level of cellulose, the dietary fiber present in the WD, as cellulose has been shown to reduce susceptibility to DSS-induced colitis by altering the composition of the microbiome [81]. Butyrate, the fermentation product from cellulose and starch [82] is suggested to support the wound healing process in IBD patients [83] and in a recent mouse study, feeding a WD reduced butyrate synthesis in the gut probably by alteration of the microbiota [84]. Moreover, recent studies suggest that there is a feedback loop between bile acids and gut microbiota [85].

Causality between microbiota and carcinogenesis has been demonstrated in gnotobiotic mice [86]. The microbiota can either be oncogenic or tumor suppressive [87]. In our study the WD changed the composition of the microbiota; interestingly after switching the diet from WD to AIN93G, the microbiota again realigned with the AIN group. Feeding WD decreased the abundance of the lactic acid producing *Faecalibaculum rodentium* [88]. Lactic acid producing bacteria are used as probiotics and can support the growth of tumor-suppressive short fatty acid-producing bacteria [89]. Additionally, WD reduced the amount of *Ruminoclostridium,* which were inversely related to colorectal cancer [90,91]. WD also decreased the Firmicutes to Bacteroidetes ratio; this ratio was correlated with obesity and adult age [92,93]. However, the association of Firmicutes to Bacteroidetes ratio with diseases is less clear. Intestinal inflammation can reduce the number of butyrate producing obligate anaerobes from the Firmicutes phylum. Nonalcoholic steatohepatitis (NASH) and IBD patients had more Bacteroides and fewer Firmicutes than healthy controls [94,95]. Microbial metabolites often serve as a link between diet and molecular signaling in the gastrointestinal tract. Our model showed that a change towards a more balanced diet leads to a less tumorigenic microbiome. 

The regenerative zone of the colonic crypt is composed of cells that have the capacity of self-renewal, survival, and proliferation and therefore are susceptible to environmental stimuli from diet and to mediators of inflammation [96,97]. Colon cancer arises from this regenerative zone [34]. In patients with IBD the crypt dynamic in this zone is disturbed and crypts show a higher rate of apoptosis [98]. The regeneration of the intestinal mucosa during the healing phase is based on the wound healing mechanism, which is dysregulated in IBD patients [99]. The regeneration is influenced by the diet, and is supported among others by vitamin D, fiber, and amino acids [100]. Butyrate, the product of fiber digestion, is essential for colonic crypt maintenance, although it can have opposing effects on colonocytes, increasing proliferation of the normal colonic crypt cells and inhibiting proliferation of colon cancer cells [101,102], as observed in our study in the mice fed the fiber rich AIN93G. Moreover, the diet switch to the AIN93G diet decreased Bax/Bcl-2 ratio, indicating less susceptibility to the pro-apoptotic effect of DSS. While the high fiber and cellulose content of the AIN93G diet favors butyrate synthesis, feeding butterfat leads to the synthesis of secondary bile acids, such as deoxycholic acid (DCA) [103] which is considered as toxic and carcinogenic [104]. In rat intestinal epithelial cells, DCA inhibited cell growth probably by counteracting EGFR-signaling [105].

On the molecular level, epithelial cell turnover and wound repair is regulated by Wnt/β-catenin and NF-κB, but unresolved inflammation may lead to their aberrant activation and to subsequent tumor promotion [106]. Although mutations in the Wnt/β-catenin genes occur late during development of CAC, several studies observed early activation due to cross activation by inflammatory cytokines [107]. In mouse models of colitis, the Wnt/β-catenin pathway was inhibited or activated depending on the stage of inflammation [108]. Enhanced regeneration in colon of Dkk1^d/d^ mice, expressing low levels of the Wnt inhibitor Dkk1, did not promote tumorigenesis in AOM/DSS treated animals [109]. Delayed mucosal regeneration with reduced expression of *Lgr5* and *C-myc* has been observed previously in a model of colitis in STAT6^−/−^ mice [110]. LGR5, located exclusively at the base of the colonic crypt, is a marker for cycling cells [56]. Peregrina et al. have demonstrated that a similar diet as the WD used in our study, high in fat but deficient in calcium, vitamin D and methyl donors reduced the Ki-67-positive cell fraction of LGR5-positive murine small intestinal stem cells despite the demonstrated tumor promoting effect of this diet [111]. In our study, the WD rather inhibited the Wnt pathway in the ascending colon, where it impaired regeneration after DSS-induced epithelial injury. Cell death was suggested to be a driver of colitis and overexpression of the pro-survival protein Bcl-2 in intestinal epithelial cells ameliorated chronic inflammation in an interleukin-10 (IL-10) knockout mouse model [112]. This is in line with the increase of *Bcl-2* in the diet switch group suggestive for a possible mechanism by which the AIN93G diet counteracts chronic colitis. The reduced mRNA levels of the Wnt target genes *C-myc*, *Bcl-2,* and *Axin2* in the WD group could be due to the lower expression of the transcription factor of the Wnt pathway *Tcf4*. *Axin2* and *Gsk-3β*, that promote degradation of β-catenin [34], were higher expressed in the AIN group, suggesting that the activity of the canonical Wnt signaling was still under control and served the physiological process of tissue regeneration. 

This study has several limitations. Although the AOM/DSS model is largely used to understand human tumorigenesis, it needs to be considered that there are large differences in the pattern of mutant genes and perturbed pathways between human and mouse colorectal tumors [113]. In our study, we used only female BALB/c mice, in order to have a more homogeneous group; therefore, the findings might be different in male mice, or in other mouse strains.

## 5. Conclusions

**Figure 8:**
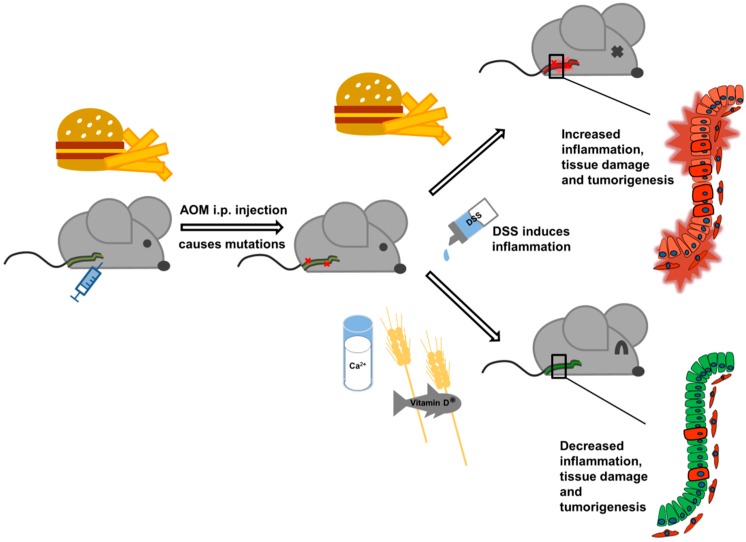
Effect of a Western Diet in the chemically-induced colorectal cancer model.

Our results demonstrate the importance of the diet for tumorigenesis in the CAC model. A WD high in butter fat and low in calcium, vitamin D, methyl donors, and fiber significantly worsened DSS-induced colitis and facilitated tumor promotion (Figure 8). Our data suggest that the WD impairs the intestinal cell turnover in yet untransformed mucosa, and through this, increases the susceptibility to inflammatory and carcinogenic stimuli leading to unresolved chronic colitis. In turn, this creates a tumor-promoting environment for already initiated intestinal epithelial cells. We could show that the change to the healthy diet after tumor initiation corrected the deleterious effect of the WD may be due to increased wound healing and prevention of chronic inflammation. This suggests that a change in dietary habits to a balanced diet, if done in time, could also have chemopreventive effects in humans, preventing or reducing disease activity and cancer risk in the colon, and could therefore contribute to a reduction of healthcare costs. 

## Figures and Tables

**Table 1:** Effect of the diet on the number of low-grade (LG) and high-grade (HG) dysplasias and of carcinomas in situ (CIS).

Group	Number of LG Dysplasia	Number of HG Dysplasia	Number of CIS
AIN	2	0	0
WD	5	1	1
WD/AIN	3	1	1

Determined in Hematoxylin-Eosin-stained cross sections of the colon (*n* = 8/group).

**Table 2:** Effect of the diet on the size of LG and HG dysplasias and of CIS.

Group	Size of LG Dysplasia (Total Number of Aberrant Crypts)	Size of HG Dysplasia (Total Number of Aberrant Crypts)	Size of CIS (Total Number of Aberrant Crypts)
AIN	78	0	0
WD	45	49	786
WD/AIN	38	27	300

Determined in HE stained cross sections of the colon (*n* = 8/group).

## Supplementary Materials

The following are available online at https://www.mdpi.com/2072-6643/12/1/45/s1, Figure S1: Body weight change at the end of the last DSS cycle relative to the day of AOM injection., Figure S2: Effect of feeding the test diets for 117 days in the naïve groups not exposed to AOM/DSS on body weight change (**A**), colon length (**B**), no. of lymph follicles in whole colon (**C**), spleen weight (**D**) and liver weight (**E**)., Figure S3: Effect of the diet on (**A**) severity of chronic inflammation and (**B**) mucosal architecture in the colon., Figure S4: Effect of diet on expression of VDR protein in (**A**) healthy or (**B**) in aberrant crypts., Table S1: Composition of the experimental diets. Table S2: List of PCR primers used in this study, Table S3: Effect of the diet on mRNA expression of genes of the Wnt pathway, proliferation and survival, intestinal barrier proteins, inflammation and the vitamin D system as well as the casr in colon ascendens (CA) and colon descendens (CD) of AOM/DSS-treated mice.

## Abbreviations

1:25-D_3_	1,25-dihydroxyvitamin D_3_;
25-D_3_	25-hydroxyvitamin D_3_;
ACF	aberrant crypt foci;
AOM	azoxymethane;
CAC	colitis-associated cancer;
CaSR	calcium-sensing receptor;
CRC	colorectal cancer;
CYP24A1	1,25-dihydroxyvitamin D_3_ 24-hydroxylase;
CYP27B1	25-hydroxyvitamin D_3_ 1α-hydroxylase;
CYP2R1	vitamin D 25-hydroxylase,
CYP3A11	cytochrome P450, family 3, subfamily A, polypeptide 11;
DSS	dextran sulfate sodium salt;
IBD	inflammatory bowel disease;
iNOS	inducible nitric oxide synthase;
LGR5	leucine-rich repeat-containing G-protein coupled receptor 5;
TCF4	T-cell factor 4;
VDR	vitamin D receptor;
WD	Western style diet.
