# Supplementary material for: Switching to a Healthy Diet Prevents the Detrimental Effects of Western Diet in a Colitis-Associated Colorectal Cancer Model
Source: Nutrients. 2019 Dec 23;12(1):45. doi: 10.3390/nu12010045 (PMC7019913; doi:10.3390/nu12010045)
Supplement: Supplementary file 1 [file nutrients-12-00045-s001.pdf]

Supplementary Figures and Tables

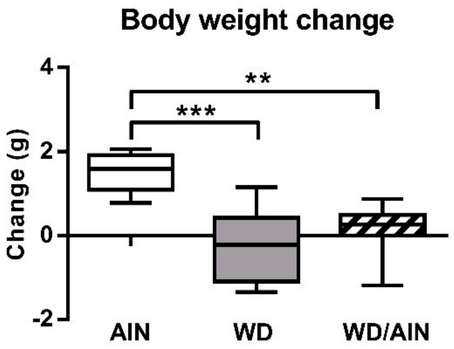

Figure S1. Body weight change at the end of the last DSS cycle relative to the day of AOM injection. Median, interquartile range and whiskers (min to max); $n = 8$; ANOVA with Tukey post-hoc test, $^{**}p < 0.01$, $^{***}p < 0.001$.

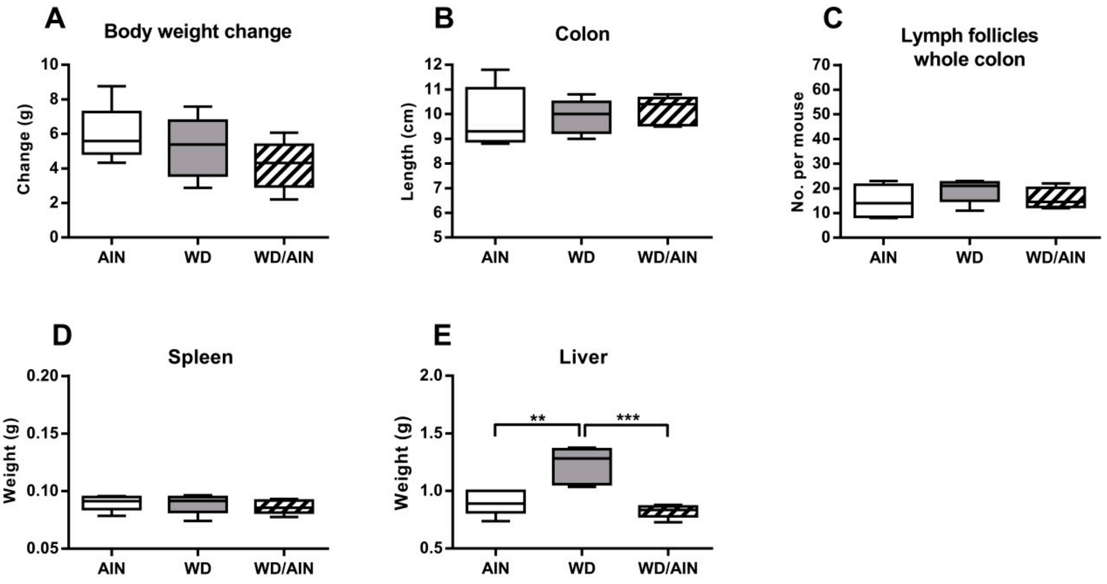

Figure S2. Effect of feeding the test diets for 117 days in the **naïve groups** not exposed to AOM/DSS on body weight change (A), colon length (B), no. of lymph follicles in whole colon (C), spleen weight (D) and liver weight (E). Median, interquartile range and whiskers (min to max); $n = 5$; ANOVA with Tukey post-hoc test, $^{**}p < 0.01$, $^{***}p < 0.001$.

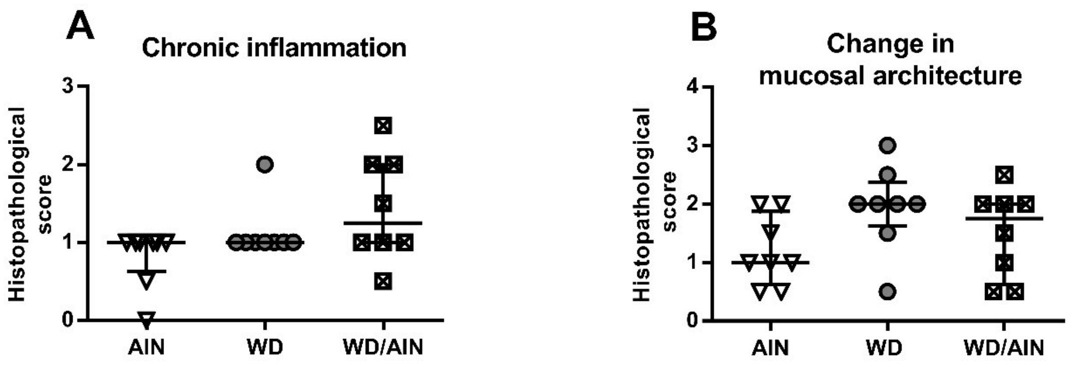

Figure S3. Effect of the diet on (A) severity of chronic inflammation and (B) mucosal architecture in the colon. n = 8; median with interquartile range, Kruskal Wallis with Dunn post-hoc test.

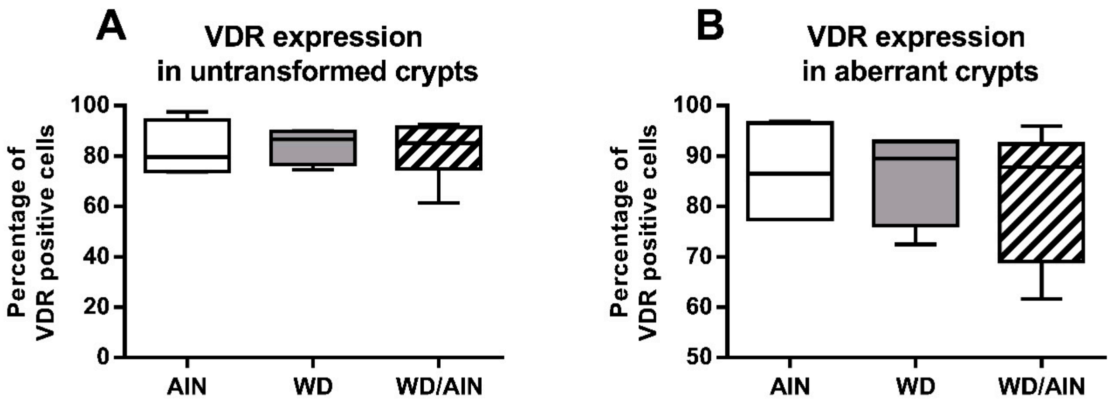

Figure S4. Effect of diet on expression of VDR protein in (A) healthy or (B) in aberrant crypts. Per group, 4-7 animals were analyzed and per mouse one area of 0.20 – 0.23 mm² was chosen manually. Median, interquartile range and whiskers (min to max), ANOVA with Tukey post-hoc test.

Table S1. Composition of the experimental diets. The values of the micronutrients refer to the supplemented amounts. Totally, the WD contained 12µg/kg vitamin D_3, the extra amount coming from the butter fat. The diets contain DL-methionine from casein.

Components	Western Diet (WD) (TD88137 SSNIFF modified)	AIN93G (control diet)
Crude Fat, g/kg	212 (butter fat)	71 (soybean oil)
Crude Protein, g/kg	173	176
Crude Fiber (cellulose), g/kg	20	50
Crude ash, g/kg	23	31
Starch, g/kg	171	382
Sugar g/kg	363	112
Calcium, g/kg	0.5	5.1
Vitamin A, IU/kg	15,000	4,000
Vitamin D_3, µg/kg	2.75	25
Vitamin E, mg/kg	150	75
Vitamin K_3, mg/kg	20	4
Vitamin C, mg/kg	1,030	-
Phosphorus (PO4), g/kg	3.6	3.2
Folic acid, mg/kg	0.23	2.1

DL-methionine, g/kg	0.0	5.2
Choline bitartrate, g/kg	1.2	2.5
Choline chloride, g/kg	0.0	1.25
Metabolizable energy MJ/kg	19.9	16.2

Table S2. List of PCR primers used in this study.

Primer	Forward	Reverse
Cyp2r1	GAGGCATATCAACTGTCGTTCT	TGGAATTGAGTAAGCCTCCCA
Cyp27a1	TCTTCATCGCACAAGGAGAG	ATAACCTCGTTTAAGGCATCC
Cyp3a11	GCCACTCACCCTGATATCCAGA	ATACGTGGGAGGTGCCTTGTT
Pxr	ACACCTGGCCGATGTGTCA	GGCAGGTCCCTAAAGTAGGATATGA
Cyp27b1	GTGTTGAGATTGTACCCTGTG	GGGAGACTAGCGTATCTTGG
Cyp24a1	GAGTCCATGAGGCTTACCC	GTGTATTCACCCAGAACGG
Vdr	GGATCTGTGGAGTGTGTGGAGACC	CTTCATCATGCCAATGTCCAC
CaSR	GAGGCCTGGCAGGTCCTGAA	TGATGGAGTAGTTCCCCACC
Dkk1	CAACTACCAGCCCTACCCT	CATAGCGTGCCTCATGCAG
Wnt3	CCGCTTCTGTCTAGGGTCTG	GGTAGAGAGTGCAGGCAAGG
Wnt5a	AATCCACGCTAAGGGTTCCT	TACTGTCCTACGGCCTGCTT
Axin2	GACCCAGTCAATCCTTATCAC	GGACTCCATCTACGCTACTG
Gsk3β	GCGATTTAAGAACCGAGAGC	CAGCACCAGGTTAAGGTAGAC
Tcf4	ACTGGCTCAAATGTAGAAGAC	GTCCCATCTCCATAGTTCCTG
Lgr5	CCTACTCGAAGACTTACCCAGT	GCATTGGGGTGAATGATAGCA
Cd44	CCATCTAGCACTAAGAGCGG	GTGTCTGGGTATTGAAAGGTG
C-myc	GTTGGAAACCCCGCAGACAG	ATAGGGCTGTACGGAGTCGT
Bcl2	CATGTGTGTGGAGAGCGTCAA	GCCGGTTCAGGTACTCAGTCA
Cyclin D1	GAACAAGCTCAAGTGGAAC	GAACTTCACATCTGTGGCA
Bax	TGGAGCTGCAGAGGATGATTG	GAAAACATGTCAGCTGCCACTC
Sucrase isomaltase	ACAGCAAGCCGAAAGAATCC	TTCACCATCATCCCAGAAGAG
Claudin 1	GATGTGGATGGCTGTCATTG	CGTGGTGTTGGGTAAGAGGT
Claudin 2	TATGTTGGTGCCAGCATTGT	TCATGCCCACCACAGAGATA
Claudin 5	GCTCTCAGAGTCCGTTGACC	CTGCCCTTTCAGGTTAGCAG
Occludin	GCTGTGATGTGTGTGAGCTG	GACGGTCTACCTGGAGGAAC
Zo1	CCACCTCTGTCCAGCTCTTC	CACCGGAGTGATGGTTTTCT
Tlr4	AGGAGTGCCCCGCTTTCACCT	CCTTCCGGCTCTTGTGGAAGCC
Cox2	AATATCAGGTCATTGGTGGAG	CAGGTTCTCAGGGATGTG
Inos	TGCCCCTTCAATGGTTGGTA	ACTGGAGGGACCAGCCAAAT
Il17a	TTTAACTCCCTTGGCGCAAAA	CTTTCCCTCCGCATTGACAC
IL6	CCTCTCTGCAAGAGACTTCCA	AGAATTGCCATTGCACAACTCT
TNF-α	TCAGCCTCTTCTCATTCCTG	CAGGCTTGTCACTCGAATTT
IFNγ	GCGTCATTGAATCACACCTG	TGAGCTCATTGAATGCTTGG
Il4	GAGCTCGTCTGTAGGGCTT	GACTCATTCATGGTGCAGC
Il10	GGTTGCCAAGCCTTATCGGA	ACCTGCTCCACTGCCTTGCT

Table S3. Effect of the diet on mRNA expression of genes of the Wnt pathway, proliferation and survival, intestinal barrier proteins, inflammation and the vitamin D system as well as the casr in colon ascendens (CA) and colon descendens (CD) of AOM/DSS-treated mice. ANOVA with Tukey *post-hoc* test (A-E) or Kruskal Wallis with Dunn *post-hoc* test, (n = 4–8, ***$p < 0.001$, **$p < 0.01$, *$p < 0.05$).

Gene of interest	CA, AOM/DSS-treated groups mRNA levels (2^-ΔΔCt, median value)			Stat. analysis CA	CD, AOM/DSS-treated groups mRNA levels (2^-ΔΔCt, median value)			Stat. analysis CD
	AIN	WD	WD/AIN		AIN	WD	WD/AIN	
**Signaling molecules of the Wnt pathway**								
Ddkk1	5.45	10.42	6.28	ns	2.03	2.60	2.73	ns
Wnt3a	3.52	4.22	5.54	ns	1.77	3.36	3.04	ns
Wnt5a					2.62	2.88	4.07	ns
Gsk3β	2.27	1.92	1.59	**AIN > WD/AIN** ***				
Axin2	5.33	3.90	4.93	**AIN > WD** **	4.79	3.56	3.65	ns
Tcf4	3.80	2.70	3.16	**AIN > WD** *	4.71	4.00	4.31	ns
**Target genes of the Wnt pathway**								
Lgr5	3.55	1.22	2.87	ns	0.15	0.33	0.11	**WD > WD/AIN** *
Cd44 x100	123.30	89.23	116.20	ns	39.59	61.48	42.74	ns
Cmyc	2.59	1.89	2.14	**AIN > WD** *	2.44	2.89	2.18	ns
***Bcl2* x100**	231.40	196.10	301.20	**WD < WD/AIN** *, **WD < WD/AIN** *	427.10	471.60	426.80	ns
Cyclin d1 x100	115.90	73.50	105.30	ns	88.17	92.84	77.22	ns
**Genes involved in apoptosis and differentiation**								
Bax	1.51	1.35	1.10	ns	1.40	1.55	1.68	ns
***Sucrase isomaltase* x100**	116.60	74.88	71.27	**AIN > WD/AIN** *, AIN > WD ns (p = 0.05)				
**Intestinal barrier proteins**								
Claudin 1 x100	86.46	54.09	77.05	ns	10.81	6.03	10.07	ns
Claudin 2	2.18	2.08	1.97	ns	1.05	0.71	0.54	ns
Claudin 5 x100	150.70	89.74	164.90	ns				
Occludin	2.59	2.24	1.84	ns	2.95	1.92	1.94	**AIN > WD/AIN** **
Zo1	4.90	5.30	4.54	ns	3.08	2.60	2.67	ns
**Genes involved in NFκB-mediated transcription**								
Tlr4	1.79	1.76	1.87	ns	2.27	2.23	1.98	**AIN > WD/AIN** *
Cox2	2.52	2.11	2.22	ns	2.09	2.06	2.56	ns
***Inos* x100**	3.00	6.02	3.71	**AIN < WD** **, **WD/AIN < WD** *	3.16	4.97	4.99	ns
**Inflammatory cytokines**								
Il17a x100					20.05	42.02	25.72	ns
Il6 x100	13.80	8.79	6.89	ns	46.50	64.82	208.80	ns
Tnf-α x100	14.28	13.06	14.16	ns	43.00	46.93	38.95	ns
Ifnγ x100	7.08	6.27	7.80	ns	9.21	14.43	12.22	ns
IL4 x100	20.10	15.93	22.45	ns	74.32	84.33	107.90	ns
I10	13.71	12.75	19.16	ns				
**Vitamin D system, Casr**								
Cyp27b1	1.83	2.01	1.61	ns	2.19	1.77	1.28	ns
***Cyp24a1* x100**	42.42	1217.00	94.41	**AIN < WD** ***, **WD/AIN < WD** ***	13.69	19.31	7.80	WD > WD/AIN ns (p = 0.07)
Vdr	4.73	3.58	4.87	ns	2.62	1.52	1.63	ns
Casr	3.63	3.47	3.07	ns	3.43	3.63	3.43	ns
